# Efficient Photocatalytic Luminous Textile for Simulated Real Water Purification: Advancing Economical and Compact Reactors

**DOI:** 10.3390/ma17020296

**Published:** 2024-01-07

**Authors:** Amin Aymen Assadi

**Affiliations:** 1 College of Engineering, Imam Mohammad Ibn Saud Islamic University (IMSIU), Riyadh 11432, Saudi Arabia; aaassadi@imamu.edu.sa or aymen.assadi@ensc-rennes.fr; 2 ENSCR, University Rennes, 11, Allée de Beaulieu, CS 50837, 35708 Rennes Cedex 7, France

**Keywords:** compacity, photocatalysis, water remediation, TiO_2_-coated luminous fabric, mineralization, modeling

## Abstract

The growing worldwide problem of wastewater management needs sustainable methods for conserving water supplies while addressing environmental and economic considerations. With the depletion of freshwater supplies, wastewater treatment has become critical. An effective solution is needed to efficiently treat the organic contaminants departing from wastewater treatment plants (WWTPs). Photocatalysis appears to be a viable method for eliminating these recalcitrant micropollutants. This study is focused on the degradation of Reactive Black 5 (*RB5*), a typical contaminant from textile waste, using a photocatalytic method. Titanium dioxide (TiO_2_) was deposited on a novel luminous fabric and illuminated using a light-emitting diode (LED). The pollutant degrading efficiency was evaluated for two different light sources: (i) a UV lamp as an external light source and (ii) a cold LED. Interestingly, the LED UV source design showed more promising results after thorough testing at various light levels. In fact, we note a 50% increase in mineralization rate when we triple the number of luminous tissues in the same volume of reactor, which showed a clear improvement with an increase in compactness.

## 1. Introduction

Drug residues and dyes are the widely reported main sources of pollution in wastewater [1]. It is described that a large proportion of polluted water goes directly to wastewater treatment plants, and one of the major problems is the growing accumulation of stable toxic substances that present chemical hazards [2]. The situation is becoming critical due to the lack of rational water treatment systems capable of reducing or eliminating the concentration of recalcitrant pharmaceutical substances in water [3]. The main problem is the way in which they are eliminated, since conventional processes (such as filtration, coagulation or adsorption) are not satisfactory in the process of eliminating various types of pollutants [4,5].

It is, therefore, necessary to develop innovative solutions that can be coupled with other processes to eliminate recalcitrant compounds [6]. At present, heterogeneous photocatalysis with TiO_2_ under UV irradiation is considered an interesting alternative, as it is a process that not only degrades the target molecules but also mineralizes the byproducts [7,8,9,10]. The advantage of using TiO_2_ lies in its commercial availability, low toxicity and photochemical stability. It is well established that TiO_2_ under UV radiation degrades and mineralizes a wide range of organic pollutants [11,12].

The current tendency is to work with the supported catalyst systems in the reactors; this eliminates the requirement for a catalyst recovery step [13]. However, UV irradiation of the catalytic surface is challenging with this sort of device, limiting the photocatalytic effectiveness of the process [14]. In the field of wastewater treatment, several different processes have been developed to address a variety of issues [15]. In recent years, several reactors have been developed to meet the needs of wastewater treatment. The fixed-bed reactor uses the diffused as well as the direct part of UV solar irradiation [16]. The main feature of this reactor is its geometric shape with a slightly inclined plate [17]. Photocatalytic membrane reactors (PMR) are one of the oldest configurations in the field of water treatment and can be classified into two distinct groups, namely suspended PMR (photoreactor with catalysts in liquid suspension) and immobilized PMR (photoreactor with catalysts immobilized in or on the membrane) [18]. Despite the many advantages of these reactors, the problem of membrane fouling remains a limiting factor in this process, as it leads to a reduction in flux and effective filtration area and shortens the membrane life. Recently, a research paper has dealt with TiO_2_ deposited on luminous textiles; the TiO_2_ substrate is illuminated by a UV LED emitter connected to a degraded optical fiber. Different types of coating were tested in this study, and the experiments were carried out in a batch reactor [19]. Indeed, the work of Elmansba and colleagues has demonstrated the effectiveness of TiO_2_ on luminous textiles in removing flumequine from wastewater [20]. In this study, two configurations of luminous textiles were used (single-sided and double-sided), and it was shown that the photocatalytic performance of the double-sided was better than that of the single-sided in ultrapure water. Various solutions have been proposed to speed up the degradation process, such as combining with other destructive processes, like ozonation, or other recovery processes like adsorption [21]. Méndez-Arriaga and colleagues [22] showed that adding O_3_/H_2_O_2_ couple to the solution improved the efficiency of the photocatalysis process, since they achieved 99% degradation and increased biodegradability after treatment. The incorporation of a solar panel and photo-Fenton reagents to the process with different reactor configurations and scales could reduce the operating cost of photocatalytic processes, and the coupling of TiO_2_ to ferrioxalate would allow the use of a pH close to neutrality, reducing costs before disposal [23]. To minimize the operating costs, the possible use of photovoltaic panels would make economic sense. However, the operating costs of such a process remain high [24,25].

In the case of suspended catalysts, the need for a separation unit limits the scaling-up process [26]. Decantation could be an alternative, but its application implies the use of bulky tanks to store the suspension. Immobilizing the catalyst on substrates as a photocatalytic coating provides advantages, namely the elimination of the separation step for µm or nm sized catalyst particles from the treated solution [27]. The main challenge to overcome in photocatalytic reactors is the diffusion of the light through the solution [28]. Reactors designed to date consider external excitation of the photocatalyst using UV lamps or natural light reaching the catalyst surface [19]. In light of existing research, the potential of luminous textiles as a viable solution to address the aforementioned challenges (cost, suspension, in situ lighting) is evident.

This study aims to investigate the degradation process of Reactive Black 5 (*RB5*: C_26_H_21_N_5_Na_4_O_19_S_6_), a common pollutant found in textile waste, using a photocatalytic approach. *RB5* is a dye that is part of the vinyl sulfone family [29]. These reactive dyes are heavily used as raw materials for many industries, including paper. *RB5* has negative effects on the environment and health. In fact, it prevents the penetration of light into environmental waters, even at low concentrations, and consequently reduces the process of photosynthesis in aqueous environments, causing a subsequent reduction in the concentration of oxygen in the water. *RB5* can cause serious health problems in humans and other living organisms [29]. The core invention entails the application of TiO_2_ to a unique luminous fabric, which was subsequently irradiated with light-emitting diodes (LEDs). The pollutant degrading efficiency was measured using two illumination configurations: an external light source and LED arrangement utilizing TiO_2_-coated bright cloth. This study presents an attempt to remove a scientific obstacle to the treatment of a dye with an economical intensive compact reactor. This new configuration has been validated in a real simulated effluent. To our knowledge, no work has been carried out on this intensive configuration of three luminous fabrics.

## 2. Materials and Methods

In this study, two different catalyst configurations were used, one with cellulose paper (external illumination) and the other with luminous tissue. These catalysts were supplied by Brochier Technologies (Lyon, France).

### 2.1. Supported Catalysts and Pollutants

**Figure 1:**
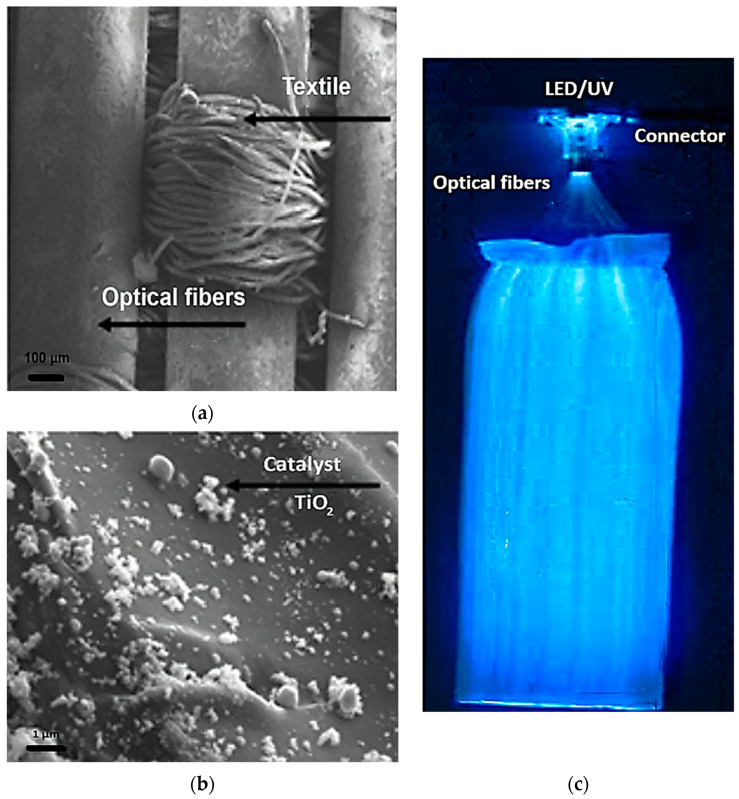
(**a**,**b**) SEM images with and without TiO_2_ and (**c**) image of optic fibers under UV light.

The luminous fabric was supplied by Brochier Technologies and it consists of polymethyl methacrylate (PMMA) optical fibers and polyester textile fibers. All the optical fibers were woven in the same direction and gathered at one end in an aluminum connector held intact by a layer of Silica. The density of TiO_2_ deposited on the fabric is about 12 g/m^2^. The new support enables pollutants to be completely decomposed while making the treatment system more compact. This installation is presented in Figure 1 with SEM images of the luminous fabric before and after the deposition of TiO_2_.

**Figure 2:**
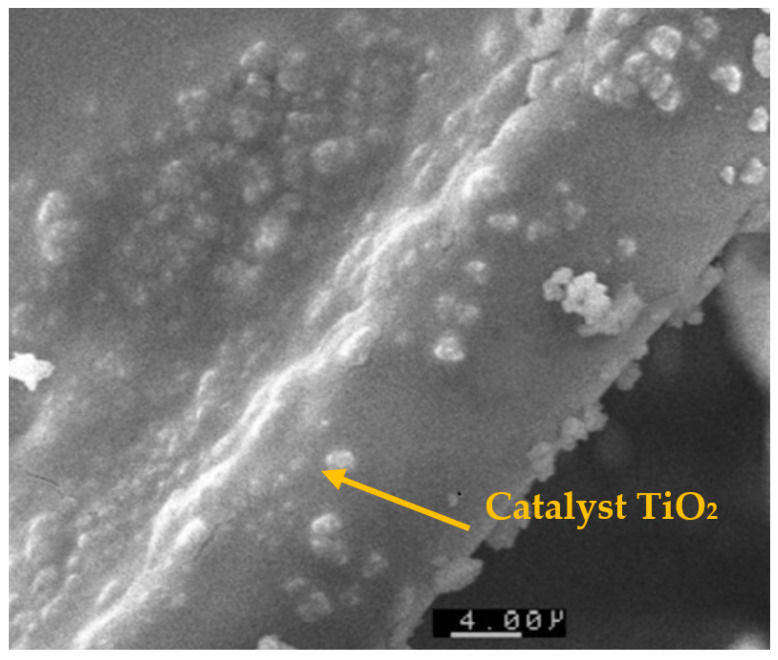
SEM image of TiO_2_ media on cellulosic support.

The cellulose paper is coated with a mixture of TiO_2_ and silica at a density of 16 g/m^2^. It is a non-woven support that limits TiO_2_ catalyst leaching from the support and ensures good photocatalytic reactivity and resistance to the UV radiation. These cellulosic paper sheets are reported in Figure 2.

These two photocatalytic media, with similar amounts of TiO_2_, were compared in terms of degradation and mineralization of *RB5*.

### 2.2. Reactors Configurations

**Figure 3:**
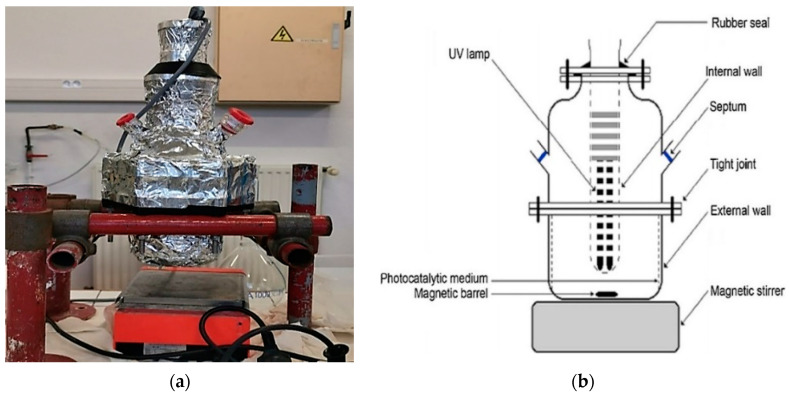

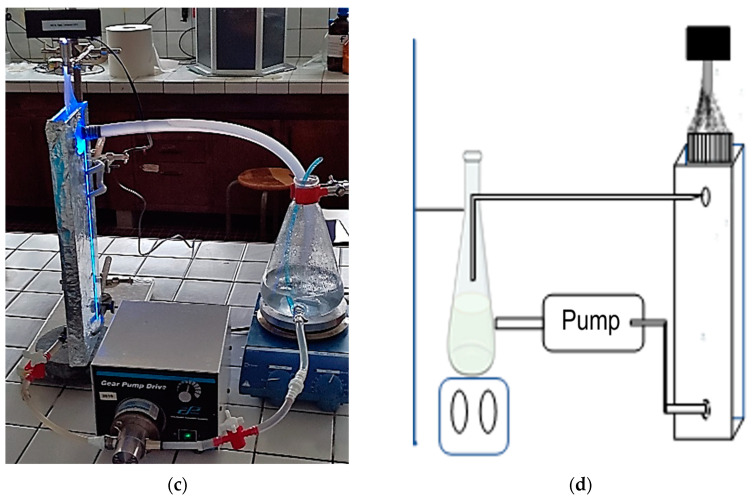
(**a**,**b**) Diagram and image of the cellulose paper reactor; (**c**,**d**) image and flow diagram of planar optical fibers supported reactor.

The first set-up shown in Figure 3a,b is a batch reactor with a capacity of 1.5 L. The solution in the batch reactor was continuously agitated using a magnetic stirrer to achieve homogenization of the liquid phase. The cellulose paper catalyst is suspended on a stainless-steel support and fixed inside the reactor.

A Philips PL-S 9W/10/4P U-shaped lamp (Montlucon, France) is immersed in the reactor. This lamp emits UV light with a wavelength of less than 385 nm. A large part of the lamp is immersed in the solution to be treated, and the reactor is entirely covered with aluminum foil.

A new continuous-flow reactor was designed with two rectangular Plexiglas plates and covered with aluminum foil to ensure that UV rays are reflected on the photocatalytic surface (Figure 3c,d). An LED with a maximum emission wavelength of 365 nm was used for all experiments., A pump was used to recirculate the solution containing the pollutant between the reactor and the tank. Measurements of the electrical energy consumed during the photocatalytic oxidation of the pollutant on each device were carried out separately using two VOLTCRAFT. ENERGY-LOGGER 4000F devices (Bralin, Poland).

### 2.3. Experimental Protocol and Analytical Tools

The UV lamps were immersed in the solution to be treated. For the luminous textile reactor, tests were carried out by immersing the luminous textile topped by the LED in the reactor. In all experiments, the adsorption–desorption equilibrium was reached before the UV lamp was turned on.

Samples were taken with a syringe at different contact times. Before injection, samples containing TiO_2_ were filtered through a Millipore filter (0.45 µm), and dye (*RB5*) concentrations were determined by UV spectrometry in the 596 nm spectral range.

#### 2.3.1. UV-Vis Absorption Spectrophotometer

The spectrometer used to analyze the *RB5* absorption spectrum is from CARY 50 Probe (California, CA, USA). This dual-beam device delivers wavelengths ranging from 200 to 1100 nm, covering the near-infrared, visible and ultraviolet ranges.

#### 2.3.2. Total Organic Carbon (TOC) Measurement

The total organic carbon is a parameter that indicates the quantity of organic pollutants in the sample to be treated. Here, we measured the remaining pollutants using the TOC-VCPH SHIMADZU meter (Kyoto, Japan). The device is equipped with an infrared detector, which signals the quantity of carbon dioxide produced as a result of the combustion of the organic matter remaining in the sample to be treated.

The results obtained were presented according to two criteria: the conversion rate (the rate of degradation) and the mineralization rate (Equations (1) and (2)). The mineralization rate describes the percentage of pollutant conversion up to the oxidation stage (CO_2_, H_2_O and inorganic ions).

(1) $$Degradation\;rate\%=\frac{\left(C_{i}-C_{f}\right)}{C_{i}}\times 100\%,$$

where $C_{i}$ and $C_{f}$ are the initial and final concentrations of *RB5*.

The following equation was used to calculate the mineralization:

(2) $$Mineralization\%=\frac{\left({TOC}_{i}-{TOC}_{f}\right)}{{TOC}_{i}}\times 100\%,$$

where ${TOC}_{i}$ and ${TOC}_{f}$ are, respectively, initial and remaining total carbon at time t and *TOC* is expressed as (mg/L).

## 3. Results and Discussion

The effect of the initial pollutant concentration, flow rate, reactor configuration, water matrix and UV intensity on photocatalytic degradation efficiency and mineralization have been studied.

### 3.1. Effect of Initial Concentration and Reactor Configuration

**Figure 4:**
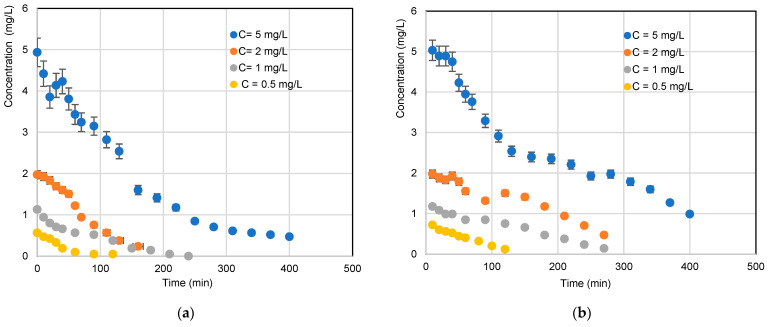
Variation in the photocatalytic degradation kinetics of *RB5* at different initial concentrations ((**a**) TiO_2_/UV/CP lamp configuration; (**b**) TiO_2_ configuration on luminous fabric).

A plot of concentration vs. time for four initial concentrations is shown in Figure 4. It can be seen that, overall, increasing the concentration has a negative effect on pollutant degradation. At lower concentrations, kinetics are faster, which can be explained by the fact that more TiO_2_ active sites are available for pollutant adsorption, and UV light reaches the catalyst surface more easily, leading to a faster rate of photocatalytic degradation. However, at high concentrations, the molecules begin to act as a screen for incident UV light. Thus, the very limited photons reached the TiO_2_ surface, leading to a decrease in photocatalytic degradation. It can also be assumed that the availability of active sites decreases with increasing concentration as a result of this screening effect. This trend is similar to that found by Dehibi and her co-workers with the photocatalytic degradation of paracetamol [2].

It is well known that in any oxidation process based on photocatalysis by TiO_2_, reactive oxygen species (ROS) such O_2_•^−^ and HO• radicals are involved in the *RB5* degradation mechanism. O_2_•^−^ and HO• radicals are produced that react and decompose the dye molecules according to the following equation of reactions [1,2,3,4,5,6,7,8,9,10]:

TiO_2_ + hυ (UVA) → TiO_2_^∗^ + e^−^ + h^+^ (3)

H_2_O + h^+^ → HO• + H^+^ (4)

O_2_ + e^−^ → O_2_•^−^ (5)

*RB5* + HO• and O_2_•^−^ → CO_2_ + H_2_O + intermediate by products (6)

**Figure 5:**
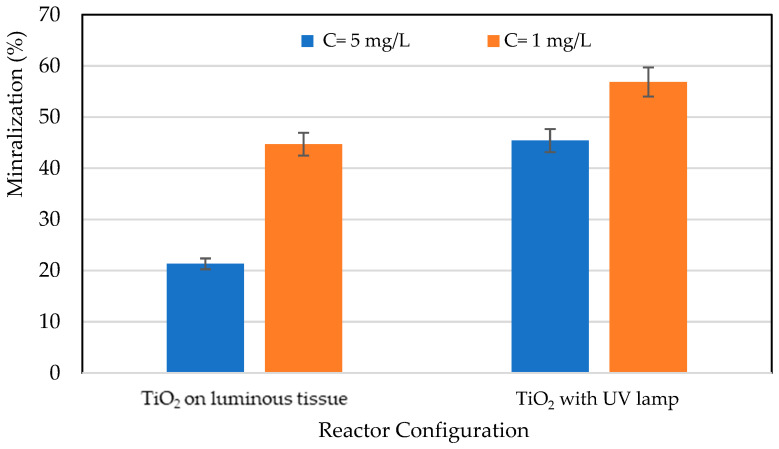
Effect of reactor configuration on the mineralization rate after total photocatalytic removal of the pollutant.

The evolution of mineralization of *RB5* was determined by measuring TOC as a function of time. Based on the results, the mineralization decreased with the increase in inlet *RB5* concentration. This trend is due to the limitation of active sites on each configuration (TiO_2_ on optical fibers or cellulosic paper). Moreover, Figure 5 shows that the reactor configuration plays a considerable role in the mineralization rate, since mineralization is high when irradiation is carried out using a UV lamp, unlike the first case without LEDs, where illumination is provided by optical fibers [8,20].

### 3.2. Performance Comparison: Cost and Compactness

To understand the degradation kinetics and adsorption process of *RB5* on the two reactors employed, the Langmuir–Hinshelwood (LH) model was used according to the following equation [30]:

(7) $$r_{0}=-\frac{d\left[RB5\right]}{dt}=k_{c}\frac{{K\left[RB5\right]}_{0}}{{1+K\left[RB5\right]}_{0}\;},$$

where *r*_0_ (mg/(L·min)): initial photocatalytic degradation rate, [*RB5*]: initial *RB5* concentration in mg/L, *K*: adsorption constant in L/mg and *k_c_*: kinetic constant mg/(L·min).

Alternatively, *k_c_* can be expressed as a function of *I* and *K*_0_ as follows:

*k_c_* = *k*_0_ × *I^δ^*, (8)

where *I* is the intensity of incident light expressed in (W/m^2^) and *δ* represents the order of intensity *I*.

The linearized LH equation is written as follows [31]:

(9) $$\frac{1}{r_{0}}=\frac{1}{kcK}\times \frac{1}{\left[RB5\right]}+\frac{1}{kc}$$

**Figure 6:**
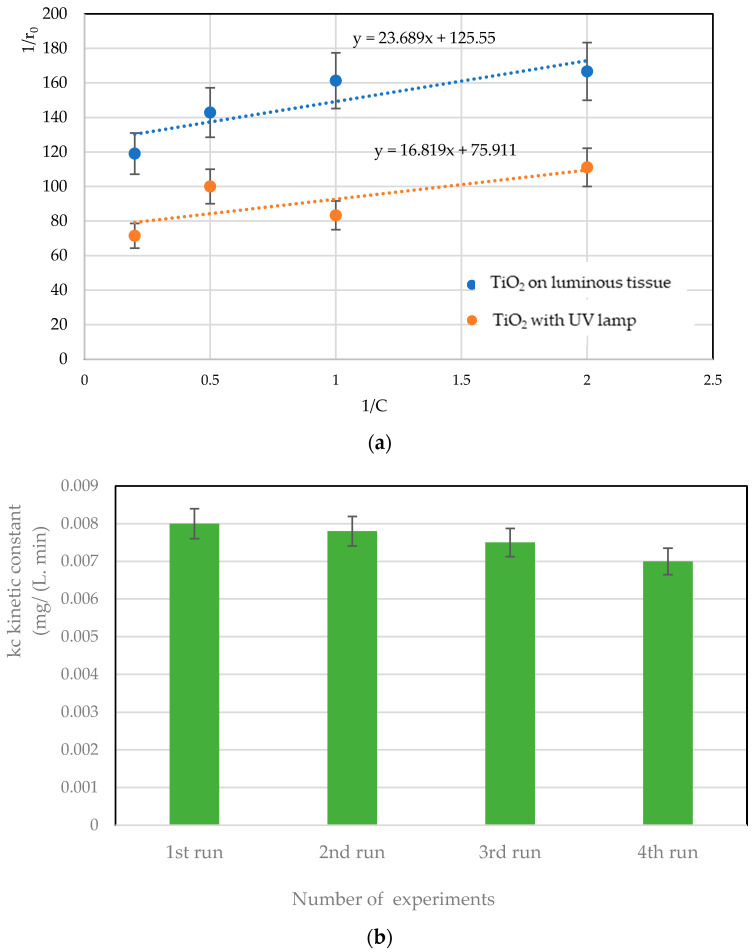
(**a**) Linear regression using the LH model for *RB5* degradation in the presence of TiO_2_ on a UV lamp. (**b**) Kinetics of reusability cycle of TiO_2_ on luminous fabric.

By plotting 1/*r_o_* as a function of 1/[*RB5*]_0_, the values of *k_c_* and *K* can be determined to visualize the shape of the straight lines, as shown in Figure 6. LH constant values are shown in Table 1.

The reuse of a supported catalyst is a crucial point in the continuous oxidation process, especially from an economic point of view, as it leads to its practical use in large-scale reactors [19,20,21]. The reusability tests of TiO_2_ on luminous fabric were carried out during an experiment of four successive cycles under similar conditions of circulation flow, mass of TiO_2_ and intensity of LED (Figure 6b). After each experiment, the catalyst was washed with ultrapure water and dried at 50 °C overnight, then used in a new experiment for 7 h. According to these tests, TiO_2_ on luminous textiles presents strong chemical stability with a very slight reduction in degradation kinetics (according to the L-H model). A reduction of less than 5% is noted after 30 h of treatment by photocatalysis.

To determine the electrical energy cost, the following equation is used [31,32,33]:

(10) $$Cost(C)=\frac{P_{e}\times t\times 1000}{V\times Disposed\;quantity(t)},$$

where P_e_ is the system input power in kW, t is the irradiation time in h and V is the volume of water in the reactor in L. The cost of 1 kwh is EUR 0.15 (in France) [34].

To determine the reactor compactness, the following equation is used:

(11) $$Compactness=\frac{Catalyst\;surface\;area\;(m^{2})}{Reactor\;volume\;including\;the\;UV\;irradition\;volume\;(m^{3})}$$

This represents a major scientific advance, especially on larger scales where pollutant transfer becomes a limiting stage in the degradation process. The choice of an optimal wastewater treatment process is highly dependent on energy efficiency [32]. Unfortunately, this criterion is most often neglected in almost all the literature comparing wastewater treatment options. However, in the case of our laboratory-scale study, an estimate of operating costs (energy and chemical costs) is reported. In the case of advanced oxidation processes, minimizing the cost of electricity would be a major advantage for the efficiency of the process.

**Figure 7:**
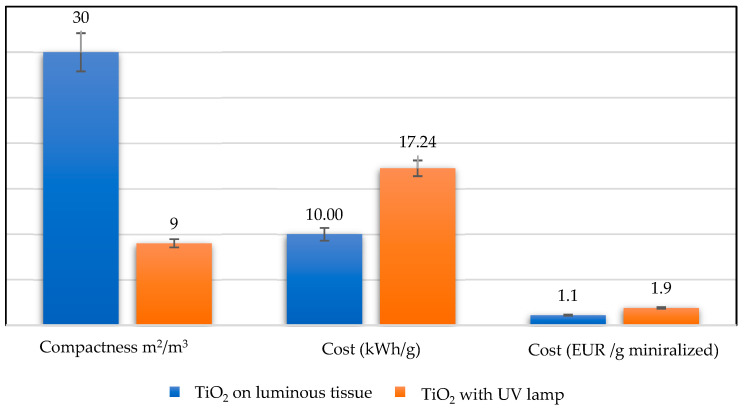
Performance evaluation criteria for the two configurations (compactness and disposal cost).

It is observed that the degradation kinetics with the TiO_2_ + UV lamp configuration were faster than with the light fabric. There is a reaction kinetic factor of 1.5 between the two configurations. Contrariwise, if the energy cost has been considered for reactor comparison, i.e., the electrical power consumed to degrade one gram of *RB5* in solution, the configuration of TiO_2_ configuration on luminous tissue presented an interesting performance (Figure 7).

This is due, on the one hand, to the in-situ illumination of the optical fiber and, on the other, to the non-negligible loss of power from the external UV lamp when heating the solution. Notably, the solution temperature was increased by 4 to 5 °C after the experiment. Similarly, with the light fabric, we were able to reduce the reactor volume by 70% compared with the TiO_2_ + UV lamp reactor [8,9,10,11,12,13,14,15,16,17,18,19,20].

Table 2 compares the expenses associated with removing organic pollutants from water using various advanced oxidation techniques. It certainly includes information on the costs of implementing these procedures, taking into consideration aspects like equipment, chemicals, energy usage and operational requirements. Based on the data presented, it appears that with one luminous tissue, the cost of eliminating organic pollution using these photocatalytic systems is comparatively similar and more favorable when compared to other advanced oxidation processes (AOPs).

This suggests that these reactors could be a low-cost approach for dealing with organic contaminants in water treatment systems.

### 3.3. Optimizing the TiO_2_ Configuration on Light Fabric

#### 3.3.1. Effect of the Number of Fabrics

**Figure 8:**
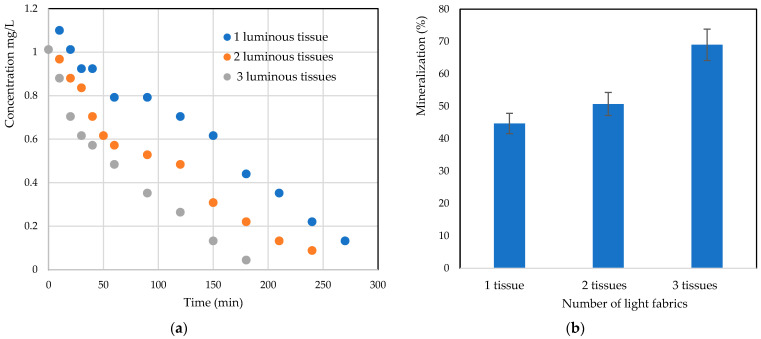
Effect of the number of luminous tissues on (**a**) *RB5* photocatalytic degradation kinetics and (**b**) mineralization efficiency.

An in-depth study was carried out to evaluate *RB5* degradation efficiency as a function of the number of luminous tissues. The final degradation efficiencies and mineralization yields obtained are shown in Figure 8a,b. It is interesting to note that the increase in the number of luminous textiles in the same volume of reactor leads to the increasing of compacity. The results obtained confirm that the degradation efficiency and mineralization were high with three light fabrics, and this trend can be confirmed in Figure 8b, where the rate of mineralization increased by 30% from one light fabric to three. This efficiency could be explained by the large catalytic surface area exposed to light when using three fabrics. Recently, work on flumequine found the same trends [20]. In fact, the increase in the amount of TiO_2_ loading has enhanced the mineralization of antibiotics.

#### 3.3.2. The Effect of UV Intensity

**Figure 9:**
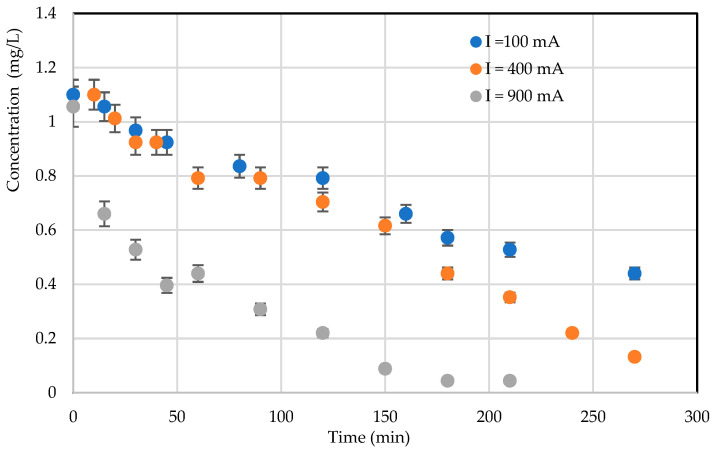
Effect of LED UV intensity on photocatalytic treatment kinetics (C = 1 mg/L; configuration: TiO_2_ on light fabric, *RB5* in distilled water).

Light intensity is an elementary factor in photocatalytic degradation, as electron-hole pairs are produced by UV light energy. Figure 9 shows the plot of degradation efficiencies with three different intensities. Different light intensities were obtained by varying the electrical intensity of the LEDs. Figure 9 shows that *RB5* degradation kinetics are faster with increasing light intensity for an initial concentration of 1 mg/L. This is explained by the fact that by increasing the light intensity, more energy reaches the TiO_2_ surface for better electron–hole pair production, and, hence, better degradation efficiencies have been obtained. It is therefore clear that the kinetic constant k increases with the light intensity.

#### 3.3.3. Recirculation Flow Effect

**Figure 10:**
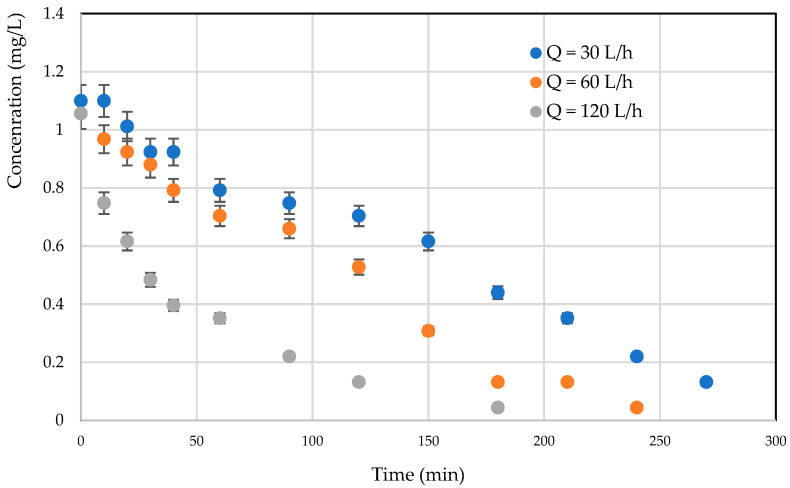
Effect of recirculation flow rate on the kinetics of photocatalytic degradation of *RB5* using TiO_2_ on luminous tissue (C = 1 mg/L: *RB5* in distilled water).

Mass transfer limitation is a key factor when studying a continuous photocatalytic process. This limitation to mass transfer can lead to a low degradation rate [35]. From a hydrodynamic point of view, a reaction involving a solid catalyst can be limited by external mass transfer. Figure 10 presents the effect of recirculation flow rate on photocatalytic degradation kinetics; it would appear that degradation kinetics are more efficient at high flow rates. It can be seen that increasing the flow rate intensifies the mass transfer step with the creation of turbulence next to the light fabric (the site of the chemical reaction). This suggests that, although the reactor is compact, the problems of transfer limitation have not yet been completely resolved [33,34,35].

#### 3.3.4. Effect of Water Matrix

To understand the feasibility of textile effluent treatment under realistic conditions, a comparison was made between effluent prepared from tap water, from EUP and from seawater (recovered from the Sillon beach—Sait Malo 35, France: temperature = 20 °C, pH~8 and salinity of ~30%). Experiments were performed with *RB5* at a concentration of 1 mg/L and a volume flow rate of 60 L/h.

**Figure 11:**
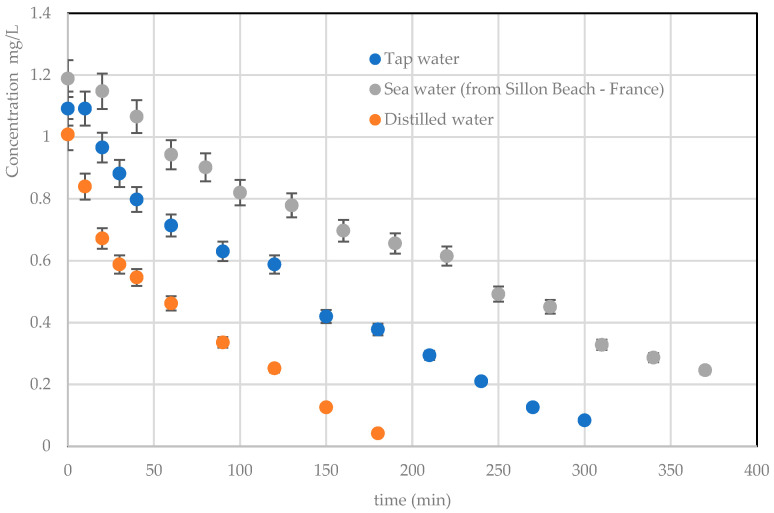
Effect of water matrices on TiO_2_ photocatalytic degradation kinetics on luminous tissue (C = 1 mg/L: Q = 120 L/h).

Figure 11 depicts the evolution of *RB5* concentration as a function of time. It can be shown that the rate of *RB5* degradation is faster in effluent prepared with EUP than in effluent prepared with tap and seawater. The presence of ions or other compounds (chloride ions, nitrates, iron, etc.) in tap water and salt water might explain this behavior. The various ions and unidentified compounds present in these waters can act as inhibitors of the photocatalytic reaction, directly influencing the rate of degradation. This is an important issue to address in this study, as we will encounter these degradation inhibitors during textile effluent treatment. Although degradation kinetics are delayed, it is interesting to note that with seawater, degradation with TiO_2_ on luminous fabric remains acceptable overall.

## 4. Conclusions

In summary, this investigation delved into the photocatalytic degradation process of the pollutant Reactive Black 5 in various water matrices like wastewater, tap water, and seawater. The result shows that the mineralization rate is increased by 50% with the increase in the number of luminous tissues in the same volume of reactor, which showed a clear improvement with an increase in compactness. Moreover, reusability tests confirm that the TiO_2_ on luminous textiles presents strong chemical stability with a very slight reduction in degradation kinetics. Throughout the study, an exhaustive assessment was conducted on diverse factors potentially impeding process efficiency, encompassing that the best configuration is that with luminous textiles in terms of *RB5* removal efficiency, mineralization and cost. This investigation underscored the advantages of the innovative configuration involving TiO_2_ deposited on light fabric substrates, primarily due to its compact form and economic viability.

## Figures and Tables

**Table 1:** Values of LH constants obtained from Figure 6.

Reactor Configuration	*k_c_* Kinetic Constant (mg/L·min)	*K*: Adsorption Constant (L/mg)
TiO_2_ on luminous fabric	0.008	4.51
TiO_2_ on cellulosic paper with UV lamp	0.013	5.30

**Table 2:** Comparison of cost of eliminating organic pollutants from water for various advanced oxidation processes.

AOP/Ref	Mineralization Rate	Operating Costs	Operating Condition
Ozonation [33]	63% of 300 mg/L	1.0 EUR ± 0.12 EUR g-TOC^−1^	Tps = 160 min pH = 12
UV/H_2_O_2_ [23]	17% of 17 mM	1.40 EUR ± 0.09 EUR g-TOC^−1^	Time = 160 min pH = 7
Photoelectric-Fenton [23,26,33]	88% with 0.1 mM of [Fe^+2^]	0.90 EUR ± 0.07 EUR g-TOC^−1^	Time = 160 min pH = 3
The present study	with only 1 luminous tissue (LT)	1.10 EUR (LT)^−1^ g-TOC^−1^	Time = 180 min pH ≈ 7

## Data Availability

The original contributions presented in the study are included in the article, further inquiries can be directed to the corresponding authors.

## References

[1] Baaloudj O., Assadi I., Nasrallah N., El A., Khezami L. (2021). Simultaneous removal of antibiotics and inactivation of antibiotic-resistant bacteria by photocatalysis: A review. J. Water Process Eng..

[2] Dhibi H., Guiza M., Bouzaza A., Ouederni A., Lamaa L., Péruchon L., Brochier C., Amrane A., Loganathan S., Rtimi S. (2023). Photocatalytic degradation of paracetamol mediating luminous textile: Intensification of the chemical oxidation. J. Water Process Eng..

[3] Timofeeva S.S., Tyukalova O.V., Timofeev S.S. (2022). Environmental risk and possibilities of ciprofloxacin phytoremediation. IOP Conf. Ser. Earth Environ. Sci..

[4] Cheikh S., Imessaoudene A., Bollinger J.-C., Hadadi A., Manseri A., Bouzaza A., Assadi A., Amrane A., Zamouche M., El Jery A. (2023). Complete Elimination of the Ciprofloxacin Antibiotic from Water by the Combination of Adsorption–Photocatalysis Process Using Natural Hydroxyapatite and TiO_2_. Catalysts.

[5] Wang G., Cheng H. (2023). Application of Photocatalysis and Sonocatalysis for Treatment of Organic Dye Wastewater and the Synergistic Effect of Ultrasound and Light. Molecules.

[6] Koe W.S., Lee J.W., Chong W.C., Pang Y.L., Sim L.C. (2020). An overview of photocatalytic degradation: Photocatalysts, mechanisms, and development of photocatalytic membrane. Environ. Sci. Pollut. Res..

[7] Selihin N.M., Tay M.G. (2022). A review on future wastewater treatment technologies: Micro-nanobubbles, hybrid electro-Fenton processes, photocatalytic fuel cells, and microbial fuel cells. Water Sci. Technol..

[8] Abidi M., Hajjaji A., Bouzaza A., Lamaa L., Peruchon L., Brochier C., Rtimi S., Wolbert D., Bessais B., Assadi A.A. (2022). Modeling of indoor air treatment using an innovative photocatalytic luminous textile: Reactor compactness and mass transfer enhancement. Chem. Eng. J..

[9] Naik S., Lee S.J., Theerthagiri J., Yu Y., Choi M.Y. (2021). Rapid and highly selective electrochemical sensor based on ZnS/Au-decorated f-multi-walled carbon nanotube nanocomposites produced via pulsed laser technique for detection of toxic nitro compounds. J. Hazard. Mater..

[10] Suhadolnik L., Pohar A., Novak U., Likozar B., Mihelič A., Čeh M. (2019). Continuous photocatalytic, electrocatalytic and photo-electrocatalytic degradation of a reactive textile dye for wastewater-treatment processes: Batch, microreactor and scaled-up operation. J. Ind. Eng. Chem..

[11] Anucha C.B., Altin I., Bacaksiz E., Stathopoulos V.N. (2022). Titanium dioxide (TiO_2_)-based photocatalyst materials activity enhancement for contaminants of emerging concern (CECs) degradation: In the light of modification strategies. Chem. Eng. J. Adv..

[12] Kang X., Liu S., Dai Z., He Y., Song X., Tan Z. (2019). Titanium dioxide: From engineering to applications. Catalysts.

[13] Fonseca-Cervantes O.R., Pérez-Larios A., Arellano V.H.R., Sulbaran-Rangel B., González C.A.G. (2020). Effects in band gap for photocatalysis in TiO2 support by adding gold and ruthenium. Processes.

[14] Ali T., Ahmed A., Alam U., Uddin I., Tripathi P., Muneer M. (2018). Enhanced photocatalytic and antibacterial activities of Ag-doped TiO_2_ nanoparticles under visible light. Mater. Chem. Phys..

[15] Theerthagiri J., Lee S.J., Karuppasamy K., Arulmani S., Veeralakshmi S., Ashokkumar M., Choi M.Y. (2021). Application of advanced materials in sonophotocatalytic processes for the remediation of environmental pollutants. J. Hazard. Mater..

[16] Akerdi A.G., Bahrami S.H. (2019). Application of heterogeneous nano-semiconductors for photocatalytic advanced oxidation of organic compounds: A review. J. Environ. Chem. Eng..

[17] Chekir N., Tassalit D., Benhabiles O., Merzouk N.K., Ghenna M., Abdessemed A., Issaadi R. (2017). A comparative study of tartrazine degradation using UV and solar fixed bed reactors. Int. J. Hydrogen Energy.

[18] Sen P., Bhattacharya P., Mukherjee G., Ganguly J., Marik B., Thapliyal D., Verma S., Verros G.D., Chauhan M.S., Arya R.K. (2023). Advancements in Doping Strategies for Enhanced Photocatalysts and Adsorbents in Environmental Remediation. Technologies.

[19] Tugaoen H.O., Garcia-Segura S., Hristovski K., Westerhoff P. (2018). Compact light-emitting diode optical fiber immobilized TiO_2_ reactor for photocatalytic water treatment. Sci. Total Environ..

[20] Almansba A., Kane A., Nasrallah N., Maachi R., Lamaa L., Peruchon L., Brochier C., Béchohra I., Amrane A., Assadi A.A. (2021). Innovative photocatalytic luminous textiles optimized towards water treatment: Performance evaluation of photoreactors. Chem. Eng. J..

[21] Baaloudj O., Nasrallah N., Kenfoud H., Bourkeb K.W., Badawi A.K. (2023). Polyaniline/Bi_12_TiO_20_ Hybrid System for Cefixime Removal by Combining Adsorption and Photocatalytic Degradation. ChemEngineering.

[22] Kutuzova A., Dontsova T., Kwapinski W. (2021). Application of TiO2-Based Photocatalysts to Antibiotics Degradation: Cases of Sulfamethoxazole, Trimethoprim and Ciprofloxacin. Catalysts.

[23] Durán A., Monteagudo J.M., Martín I.S. (2018). Operation costs of the solar photo-catalytic degradation of pharmaceuticals in water: A mini-review. Chemosphere.

[24] Surenjan A., Pradeep T., Philip L. (2019). Application and performance evaluation of a cost-effective vis- LED based fluidized bed reactor for the treatment of emerging contaminants. Chemosphere.

[25] Furukawa M., Iwamoto D., Inamori K., Tateishi I., Katsumata H., Kaneco S. (2023). Synthesis of Tungsten-Modified Sn_3_O_4_ through the Cetyltrimethylammonium Bromide-Assisted Solvothermal Method for Dye Decolorization under Visible Light Irradiation. Catalysts.

[26] Baaloudj O., Badawi A.K., Kenfoud H., Benrighi Y., Hassan R., Nasrallah N., Assadi A.A. (2022). Techno-economic studies for a pilot-scale Bi_12_TiO_20_ based photocatalytic system for pharmaceutical wastewater treatment: From laboratory studies to commercial-scale applications. J. Water Process Eng..

[27] Sansotera M., Kheyli S.G.M., Baggioli A., Bianchi C.L., Pedeferri M.P., Diamanti M.V., Navarrini W. (2019). Absorption and photocatalytic degradation of VOCs by perfluorinated ionomeric coating with TiO_2_ nanopowders for air purification. Chem. Eng. J..

[28] Indermühle C., Puzenat E., Dappozze F., Simonet F., Lamaa L., Peruchon L., Brochier C., Guillard C. (2018). Photocatalytic activity of titania deposited on luminous textiles for water treatment. J. Photochem. Photobiol. A Chem..

[29] Tang M., Li X., Deng F., Han L., Xie Y., Huang J., Chen Z., Feng Z., Zhou Y. (2023). BiPO_4_/Ov-BiOBr High-Low Junctions for Efficient Visible Light Photocatalytic Performance for Tetracycline Degradation and H_2_O_2_ Production. Catalysts.

[30] Lee K.M., Lai C.W., Juan J.C. (2018). Stability of custom-designed photoreactor for photocatalytic oxidation of Reactive Black 5 dye using zinc oxide. Corros. Eng. Sci. Technol..

[31] Garcia B.B., Lourinho G., Romano P., Brito P.S.D. (2020). Photocatalytic degradation of swine wastewater on aqueous TiO2 suspensions: Optimization and modeling via Box-Behnken design. Heliyon.

[32] Gogate P.R. (2020). Improvements in Catalyst Synthesis and Photocatalytic Oxidation Processing Based on the Use of Ultrasound. Top. Curr. Chem..

[33] Mousset E., Loh W.H., Lim W.S., Jarry L., Wang Z., Lefebvre O. (2021). Cost comparison of advanced oxidation processes for wastewater treatment using accumulated oxygen-equivalent criteria. Water Res..

[34] Assadi A.A., Bouzaza A., Wolbert D. (2016). Comparative study between laboratory and large pilot scales for VOC’s removal from gas streams in continuous flow surface discharge plasma. Chem. Eng. Resear. Des..

[35] Lou W., Kane A., Wolbert D., Rtimi S., Assadi A.A. (2017). Study of a photocatalytic process for removal of antibiotics from wastewater in a falling film photoreactor: Scavenger study and process intensification feasibility. Chem. Eng. Process. Process Intensif..

